# Use of Pyrogallol Red and Pyranine as Probes to Evaluate Antioxidant Capacities towards Hypochlorite

**DOI:** 10.3390/molecules18021638

**Published:** 2013-01-28

**Authors:** Fernanda Pérez-Cruz, Contanza Cortés, Elias Atala, Pamela Bohle, Francisco Valenzuela, Claudio Olea-Azar, Hernán Speisky, Alexis Aspée, Eduardo Lissi, Camilo López-Alarcón, Raquel Bridi

**Affiliations:** 1 Facultad de Ciencias Químicas y Farmacéuticas, Universidad de Chile, Santiago, Chile; E-Mails: ferperezc@ug.uchile.cl (F.P.-C.); colea@uchile.cl (C.O.-A.); 2 Departamento de Farmacia, Facultad de Química, Pontificia Universidad Católica de Chile, C.P. 782 0436, Santiago, Chile; E-Mails: czcortes@gmail.com (C.C.); eatala@uc.cl (E.A.); pamela.bohle@gmail.com (P.B.); fjvalen2@uc.cl (F.V.); 3 Instituto de Nutrición y Tecnología de los Alimentos, Universidad de Chile, Santiago, Chile; E-Mail: hspeisky@inta.cl; 4 Facultad de Química y Biología, Universidad de Santiago de Chile, Santiago, Chile; E-Mails: alexis.aspee@usach.cl (A.A.); eduardo.lissi@usach.cl (E.L.)

**Keywords:** pyrogallol red, pyranine, hypochlorite, antioxidant activity, plant extracts

## Abstract

Hypochlorite is a strong oxidant able to induce deleterious effects in biological systems. The goal of this work was to investigate the use of PGR and PYR as probes in assays aimed at evaluating antioxidant activities towards hypochorite and apply it to plant extracts employed in Chilean folk medicine. The consumption of PGR and PYR was evaluated from the decrease in the visible absorbance and fluorescence intensity, respectively. Total phenolic content was determined by the Folin Ciocalteau assay. PGR and PYR react with hypochlorite with different kinetics, being considerably faster the consumption of PGR. Different stoichiometric values were also determined: 0.7 molecules of PGR and 0.33 molecules of PYR were bleached per each molecule of added hypochlorite. Both probes were protected by antioxidants, but the rate of PGR bleaching was too fast to perform a kinetic analysis. For PYR, the protection took place without changes in its initial consumption rate, suggesting a competition between the dye and the antioxidant for hypochlorite. Plant extracts protected PYR giving a PYR-HOCl index that follows the order: *Fuchsia magellanica ≈ Marrubium vulgare ≈ Tagetes minuta > Chenopodium ambrosoides ≈ Satureja montana > Thymus praecox*. Based on both the kinetic data and the protection afforded by pure antioxidants, we selected PYR as the best probe. The proposed methodology allows evaluating an antioxidant capacity index of plant extracts related to the reactivity of the samples towards hypochlorite.

## 1. Introduction

In the last two decades, antioxidant capacity assessments for phytochemicals present in natural products able to inhibit (or delay) damage induced by hypochlorite have attracted the attention of several research groups. HOCl plays a relevant role in the defense mechanisms involved in the immune response towards microorganisms. Nonetheless, it has also documented that HOCl, in some patho-physiological conditions, can damage macromolecules such as proteins, DNA, RNA and cell membrane lipids, altering their biological function [1]. In particular, in the first case the ability of HOCl to react with lysine residues of proteins forming chloramine derivatives which could extended the original oxidative HOCl-mediated modifications is well-known [2]. In this context, it is important to evaluate the potential role of antioxidant compounds for minimizing the deleterious effects of hypochlorite on biomolecules and/or cellular structures, and therefore decrease its impact on human health [3]. Among the different approaches that have been developed to evaluate the scavenging activity of pure antioxidants and their complex samples towards hypochlorite, the most frequently employed strategy is based on the ability of antioxidants to protect molecular targets (proteins or other molecules). For instance, α1-antiproteinase, myoglobin and serum albumins have been used as protein targets [4,5,6,7,8,9,10]. In addition, 5-thio-2-nitrobenzoic acid (TNB), 5-aminosalycilic acid, fluorescein and luminol have been employed as hypochlorite-oxidizable target molecules [11,12,13,14,15].

It has been shown that pyrogallol red (PGR) and pyranine (PYR) dyes react efficiently with reactive species [16,17,18,19]. The spectroscopic evaluation of the bleaching of these target molecules induced by peroxyl radicals has permitted the development of assays aimed at evaluating the antioxidant capacity of pure antioxidants, plants extracts, beverages and human fluids [18,20,21,22,23,24,25]. However, the factors determining the level of protection estimated from the use of these two probes are different. While the PGR-based assay would provide an index related to the reactivity of the samples towards peroxyl radicals, the PYR-based index would be more associated with the stoichiometry of the sample-peroxyl radical reaction. In this context, it has been proposed that the use of both probes gives complementary information on the total content of antioxidants present in a complex mixture and the rate of their reaction towards peroxyl radicals [23]. In addition, PGR has also been employed as probe to estimate the scavenging activity of antioxidants present in human plasma towards hypochlorite [26]. Nonetheless, until now the antioxidant capacity of polyphenols and their complex mixtures towards hypochlorite employing this probe has not been reported. Therefore, in the present work we studied the feasibility of using PGR and PYR as hypochlorite-sensitive probes to assess the oxidant removal capacity of pure polyphenols and methanolic extracts of six plant species (*Satureja montana* L. (Lamiaceae), *Fuchsia magellanica* Lam. (Onagraceae) *Thymus praecox* subsp. *arcticus* (Durand) Jalas (Lamiaceae), *Marrubium vulgare* L. (Lamiaceae), *Tagetes minuta* L. (Asteraceae) and *Chenopodium ambrosioides* L. (Amaranthaceae). These plants were selected because they are commonly used in Chilean folk medicine as analgesic, anti-inflammatory and antispasmodic agents.

## 2. Results and Discussion

### 2.1. Interaction of PGR and PYR with Hypochlorite

**Figure 1:**
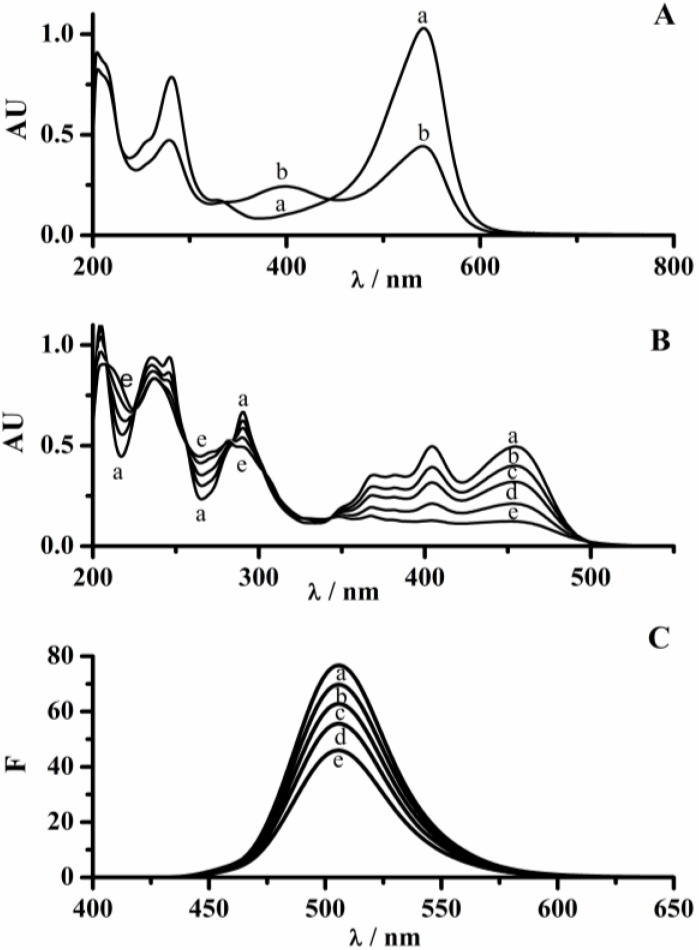
Probe spectroscopic changes induced by HOCl. (**A**) Changes in the UV-visible spectrum of PGR (30 µM) induced by hypochlorite (15 µM). Line *a* and *b* before and after 5 min of hypochlorite addition, respectively; (**B**) Changes in the UV-visible spectrum of PYR (50 µM) mediated by 100 µM of hypochlorite. Lines *a*–*e*: increasing times up to 30 min of incubation; (**C**) Changes in the fluorescence spectrum of PYR (5 µM) mediated by hypochlorite (10 µM). Lines *a*–*e*: increasing times up to 30 min of incubation.

Figure 1A and B show the effect of hypochlorite on the UV-visible spectrum of PGR and PYR, respectively. The absorption intensity of the visible band of PGR (with a maximum at 540 nm) decreased immediately (less than 10 s) after the addition of hypochlorite in a concentration-dependent way. In addition, a new band at 395 nm was generated implying that PGR was efficiently oxidized by hypochlorite. As shown in Figure 1B, the presence of hypochlorite also generated changes in the UV-visible spectrum of PYR. In fact, the absorbance at 460 nm clearly decreased while formation of a new band at 265 nm is observed. In addition, the intensity of PYR fluorescence (460 and 510 nm as excitation and emission wavelengths, respectively) decreased during its incubation with hypochlorite (Figure 1C). No changes in the shape of the fluorescence band were observed up to 60% of PYR disappearance.

**Figure 2:**
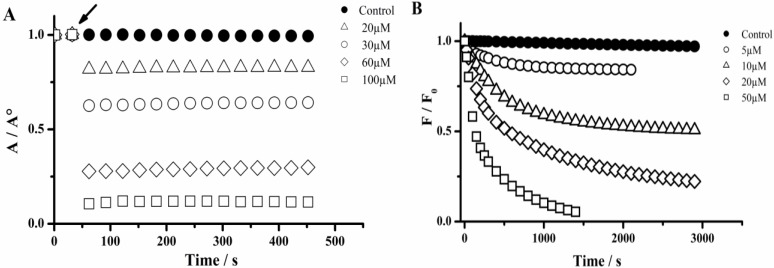
Kinetic profiles of PGR and PYR consumption induced by hypochlorite. (**A**) Consumption of PGR followed by visible spectroscopy at 540 nm. PGR (60 µM) was incubated with hypochlorite at 20; 30; 60; and 100 µM. The arrow indicates the time in which hypochlorite was added; (**B**) PYR consumption followed by fluorescence technique (460 and 510 nm for λ_ex_ and λ_em_, respectively). PYR (5 µM) was incubated with hypochlorite at 5; 10; 20; and 50 µM. Controls experiments (PGR or PYR solution in the absence of hypochlorite).

In comparison with PGR, the hypochlorite-mediated UV-visible and fluorescence changes of PYR were significantly slower. In fact, as depicted in Figure 2, different kinetic profiles were observed during the reaction of PGR and PYR with hypochlorite. In the case of PGR (Figure 2A), a fast decrease of the absorption units at 540 nm was evidenced immediately (less than 5 s) after the addition of hypochlorite. 

**Figure 3:**
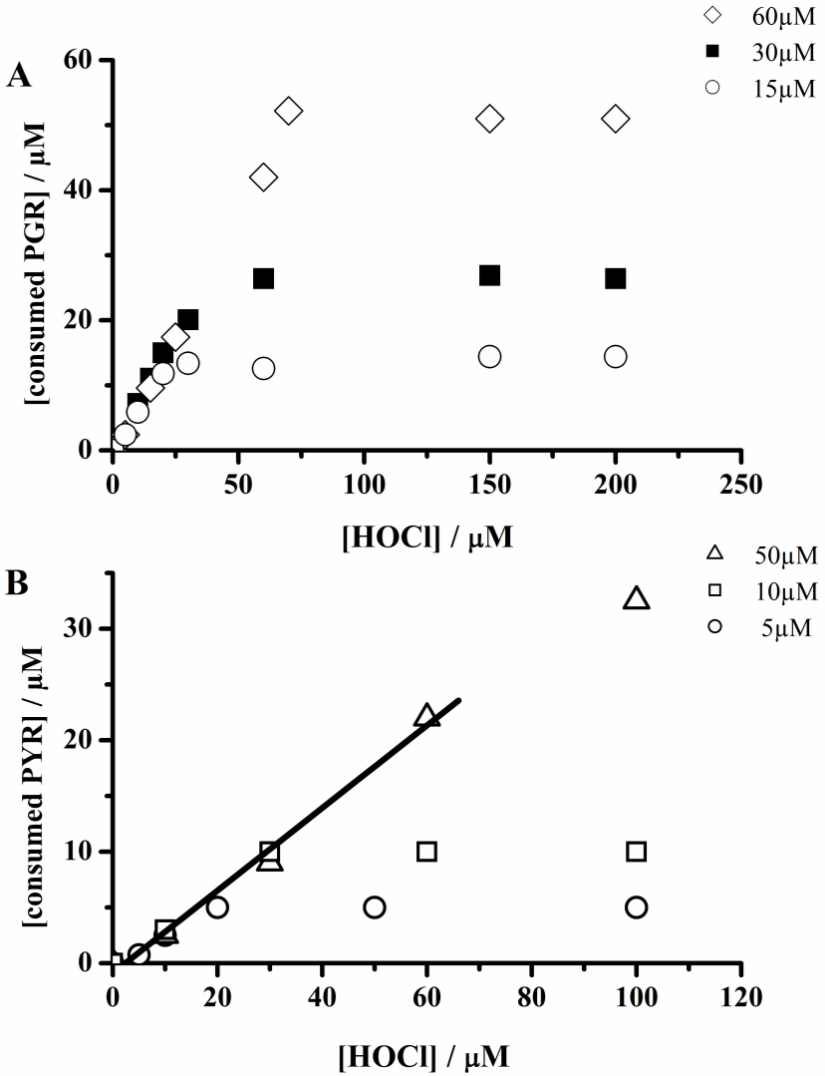
Dependence of the consumed probe with hypochlorite concentration. (**A**) Dependence of the PGR consumed with hypochlorite concentration. [PGR] = 15; 30; and 60 µM. (**B**) Dependence of PYR consumed with hypochlorite concentration. [PYR] = 5; 10; and 50 µM.

Therefore, the kinetic profiles regarding the PGR consumption induced by hypochlorite did not allow estimating the initial rate of the reaction and the hypochlorite removing capacity was characterized by the presence of a plateau in the absorbance (Figure 2A). Contrary to this behavior, the PYR-hypochlorite reaction was slow enough to allow the evaluation of the initial PYR consumption rate. Interestingly, as in the case of PGR, the kinetic profiles of PYR consumption also reached a plateau in a hypochlorite-concentration dependent way (Figure 2B) attributable to the total consumption of the oxidant. The presence of this plateau allows then an evaluation of the PGR and PYR consumed by each mol of hypochlorite added to the working solution. Then, from the dependence of consumed moles of PGR and PYR on hypochlorite concentration (Figure 3) we estimated the stoichiometry of the reaction (n) of both probes towards hypochlorite. The consumption of PGR and PYR showed a linear dependence between 1 and 20 µM of hypochlorite concentrations. In this range of hypochlorite concentrations was estimated a stoichiometry (defined as mole of probe consumed by each mole of hypochlorite) of the reaction of 0.7 and 0.33, for PGR and PYR, respectively. 

**Figure 4:**
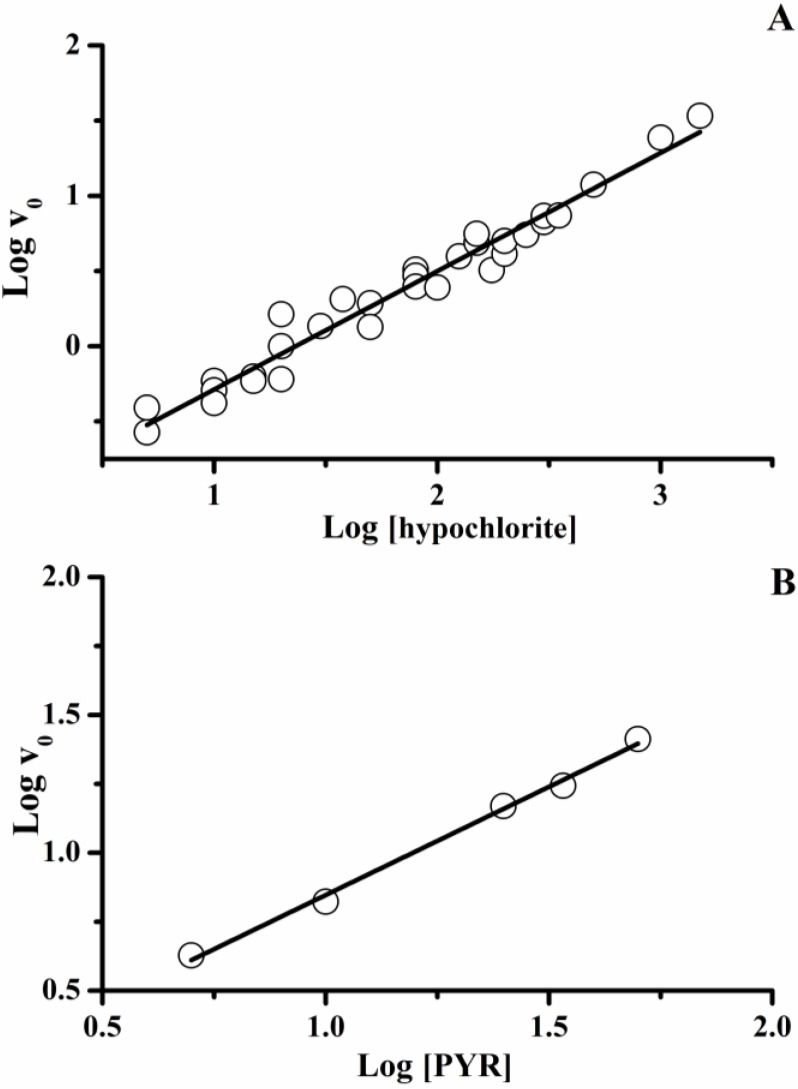
Dependence of initial consumption rate of PYR with hypochlorite (**A**) or PYR initial concentrations (**B**). Log-Log plots.

The initial consumption rate of PYR was estimated upon a wide PYR and hypochlorite concentration range, and represented as Log-Log plots in Figure 4. The data depicted in this figure gave a linear regression equation of y = 0.79x – 1.07 (r^2^ = 0.963) for the Log v_0 *versus*_ Log [hypochlorite] plot (Figure 4A), and y = 0.79x + 0.062 (r^2^ = 0.995) for the Log v_0 *versus*_ Log [PYR] plot (Figure 4B). From these results, it was estimated a kinetic hypochlorite and PYR order near to 0.8. These kinetic orders are compatible with a bimolecular process in the first stages of the reaction and the *n* value (*n* = 0.33) would reflect the presence of secondary reactions between PYR-oxidized products and hypochlorite.

### 2.2. Protection of PGR and PYR by Antioxidants

From the results presented in Figure 1, Figure 2, Figure 3, Figure 4 we chose the more appropriate experimental conditions to carry out experiments aimed to study the effect of pure antioxidants and plant extracts on the hypochlorite-mediated PGR and PYR consumption. We selected 10 µM hypochlorite concentration, and 15 and 5 µM for PGR and PYR concentrations, respectively. These experimental conditions were chosen considering near a half consumption of the probes induced by the above mentioned hypochlorite concentration. 

### 2.3. Protection by Antioxidants

**Figure 5:**
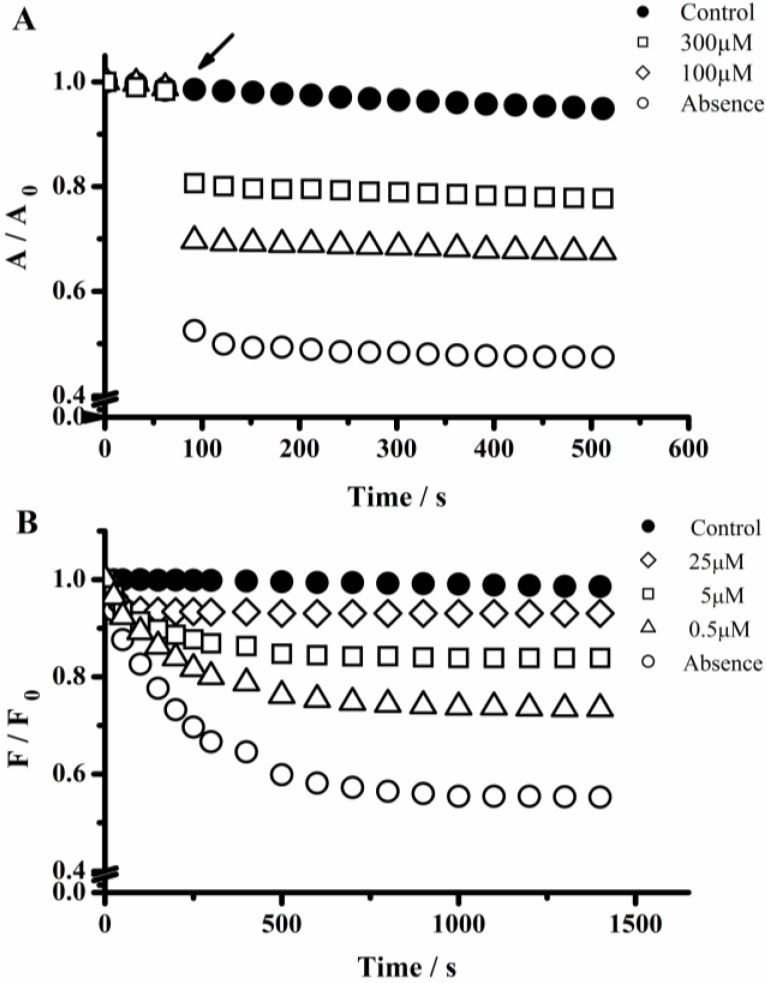
Effect of ferulic acid on the consumption of PGR or PYR induced by hypochlorite. (**A**) Kinetic profiles of the reaction between PGR (15 µM) and hypochlorite (10 µM) in the absence and presence of ferulic acid at 100 and 300 µM. The arrow indicates the time at which hypochlorite was added. (**B**) Kinetic profiles of the reaction between PYR (5 µM) and hypochlorite (10 µM) in the absence and presence of ferulic acid at 0.5; 5 and 25 µM. Controls experiments (PGR or PYR plus ferulic acid in the absence of hypochlorite).

The consumption of PGR and PYR induced by hypochlorite is inhibited by compounds able to react with this reactive species. Figure 5 shows representative results regarding the protection of PGR and PYR afforded by antioxidants. As depicted in this figure, the presence of ferulic acid clearly inhibited the consumption of both probes. In the case of PGR (Figure 5A), the protection was evidenced at higher ferulic acid concentrations (50 and 300 µM) than PGR, implying a lower reactivity of the antioxidant than PGR towards hypochlorite. Ferulic acid also protected PYR even at concentrations similar to those of the dye, 0.5–25 µM (Figure 5B). Interestingly, the kinetic data associated with the effect of ferulic acid on the PYR consumption highlights the fact that the initial consumption rate of PYR was similar in the absence and presence of the additive (even at the highest concentration employed). The latter evidences that the protection to PYR given by ferulic acid is mainly related to the direct competition by hypochlorite, disregarding possible reactions of the antioxidant with hypochlorite-generated intermediates. The same behavior was observed for all the antioxidants studied in this work.

Considering a direct competition between PGR or PYR and antioxidants towards hypochlorite, a simple competitive scheme where both processes follow the same kinetic law leads to:

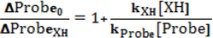
(1)
where ∆Probe_0_ and ∆Probe_XH_ correspond to the difference of the initial absorbance or fluorescence intensity of PGR or PYR and the absorbance or fluorescence intensity after the reaction with hypochlorite in absence (∆Probe_0_) and in the presence (∆Probe_XH_) of the antioxidant. k_Probe_ and k_XH_ represent the specific kinetic rate constant of the hypochlorite-probe and hypochlorite-XH reaction, respectively. [XH] and [Probe] are the initial antioxidant and PGR or PYR molar concentrations, respectively. 

**Figure 6:**
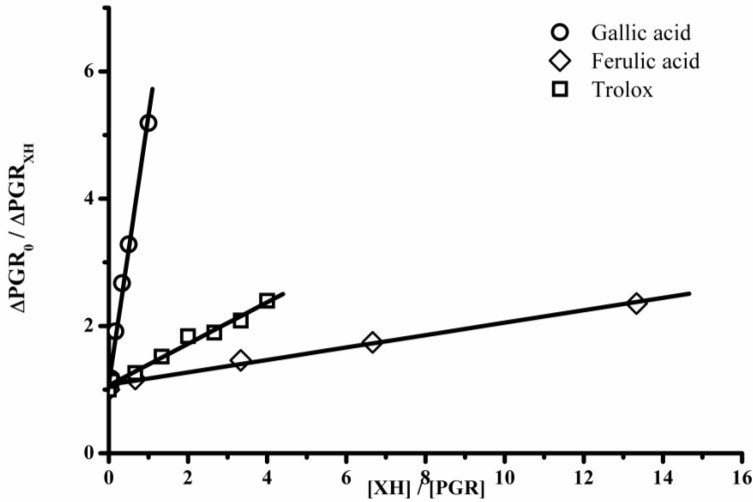
Dependence of ∆PGR_0_/∆PGR_XH_ with the [XH]/[PGR] ratio. XH = Trolox, gallic acid, and ferulic acid.

Therefore the slope of the ∆PGR_0_/∆PGR_Probe *versus*_ [XH]/[Probe] plots is directly related to the kinetic rate constant of the reaction between XH and hypochlorite and, as consequence, such slope could be considered as an antioxidant capacity index of a particular additive towards hypochlorite. Figure 6 shows typical results in which PGR was used as probe and Trolox, gallic and ferulic acid were employed as antioxidants. As is expected from Eqn. (1), a linear behavior was observed. Obtained data for Trolox, gallic and ferulic acids gave mean linear regressions of y = 1.07 − 0.32x (r^2^ = 0.991); y = 1.08 − 4.21x (r^2^ = 0.995); and y = 1.07 − 0.097x (r^2^ = 0.995), respectively. The slope of these data represents the k_XH_/k_probe_ ratio. From these type of data, employing both probes, the slopes for different antioxidants were estimated and represented as gallic acid equivalents, named as PGR-HOCl and PYR-HOCl indexes, when PGR and PYR were employed as probes, respectively. As presented in Table 1, coumaric acid was the compound with the lowest antioxidant ability, 0.004 and 0.02 gallic acid equivalents, by PGR-HOCl and PYR-HOCl assays, respectively. Quercetin was the antioxidant compound that showed the highest antioxidant ability, 4.35 and 1.5 for PGR-HOCl and PYR-HOCl, respectively. These results show that the PGR-HOCl index of quercetin was 1088 times higher than that of coumaric acid. The same comparison, but employing PYR as probe (PYR-HOCl) showed an antioxidant ability of quercetin 75 times higher than that of coumaric acid. In agreement with previous reports, which employed human serum albumin (HSA) as hypochlorite-target, coumaric acid showed a lower antioxidant capacity than caffeic, ferulic and sinapic acid [7]. In addition, the higher antioxidant capacity of quercetin towards hypochlorite than cinnamic acids has also been reported by Firuzzi *et al.* [7,8] who obtained a quercetin/coumaric acid ratio close to 900.

**Table 1:** PGR-HOCl and PYR-HOCl values (relative to gallic acid activity) of pure antioxidants. Data represent the mean of at least three independent experiments. Standard deviation (SD) represented less than 10% of the mean.

Compound	PGR-HOCl	PYR-HOCl
Trolox ^®^	0.07 ± 0.001	0.04 ± 0.003
Cafeic acid	0.01 ± 0.0008	0.70 ± 0.06
Ferulic acid	0.02 ± 0.0012	0.19 ± 0.01
Sinapic acid	0.01 ± 0.0009	0.24 ± 0.02
Cumaric acid	0.004 ± 0.0002	0.02 ± 0.001
Gallic acid	1	1
Quercetin	4.35 ± 0.30	1.5 ± 0.14
Apigenin	0.46 ± 0.04	0.56 ± 0.03
Ascorbic acid	0.44 ± 0.03	0.06 ± 0.003

Initial reaction rates can be obtained only when PYR was employed as probe. In addition, the stoichiometry of the reaction was different depending on the target molecule employed—0.7 and 0.33 moles of PGR and PYR were consumed per each mol of hypochlorite, respectively. In the case of PYR, the stoichiometric value would involve the occurrence of secondary reactions after the first step, which directly depends on the PYR and hypochlorite concentrations. When each probe was incubated with hypochlorite in the presence of antioxidants, the results obtained showed a low protection of PGR afforded by cinnamic acids. Coumaric acid, a cinnamic acid derivative with the lowest antioxidant activity, showed a PGR protection five times lower than ferulic acid (the cinnamic acid derivative with the highest ability to protect PGR). When PYR was used as probe, coumaric acid presented an antioxidant activity 35 times lower than caffeic acid (the cinnamic acid derivative with the highest activity to protect PYR). Thus, the use of PYR implies a higher discrimination than PGR in terms of the antioxidant activity of pure compounds. Therefore, and taking into account the kinetic profiles of the PYR consumption, we selected this molecule as hypochlorite-oxidable probe. The above presented results show that PYR presents advantages in comparison with PGR. Firstly, the low rate of the PYR-HOCl reaction allows estimating initial reaction rates, obtaining kinetic data regarding its consumption induced by HOCl. Secondly, the protection given by pure antioxidants follows the expected order in comparison with the activity of these compounds towards hypoclorite [7,8]. Then, we applied a competitive assay employing PYR as target to evaluate the antioxidant activity of plant extracts towards hypochlorite. 

### 2.4. Protection of PYR by Plant Extracts

**Figure 7:**
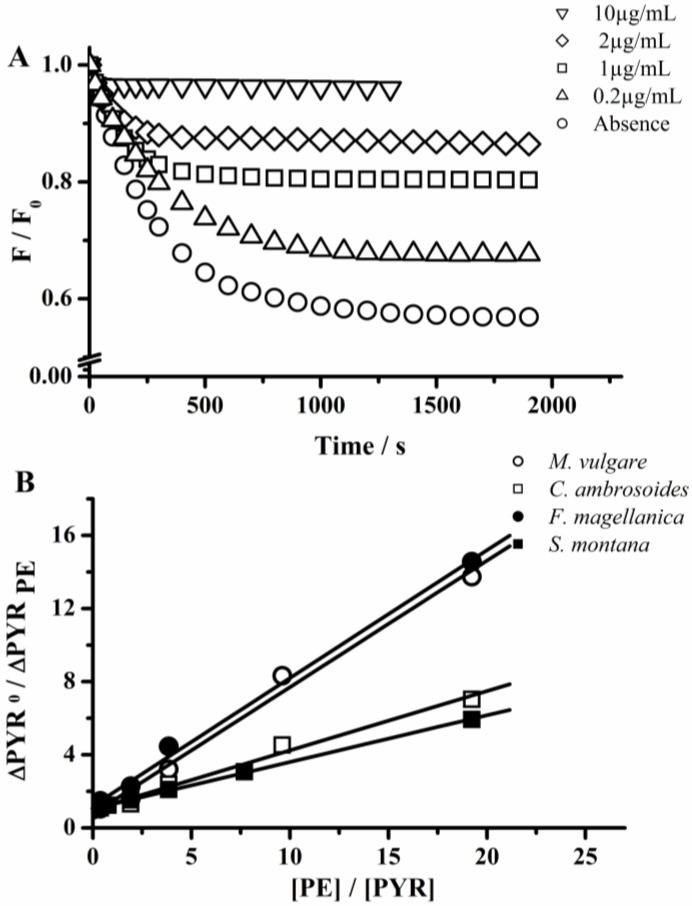
Effect of plant extracts (PE) on the hypochlorite-mediated consumption of PYR. (**A**) Kinetic profiles of the reaction between PYR (5 µM) and hypochlorite (10 µM) in the absence and presence of *F. magellanica* at 0.2; 1; 2 and 10 µg/mL. (**B**) Dependence of ∆Probe_0_/∆Probe_PE_ with [PE]/[PYR] ratio. *F. magellanica*; *M. vulgare*; *C. ambrosoides*; and *S. montana*. PE concentrations correspond to µg of dried extract/mL.

The antioxidant activity of *Marrubium vulgare* [27,28], *Tagetes minuta* [29], *Thymus praecox* [30,31] and *Satureja montana* [32,33,34] have been reported previously. The published data referred to the scavenging activity of essential oils, and aqueous or alcoholic extracts of these plants towards the 2,2-diphenyl-1-picrylhydrazyl (DPPH) free radical. This antioxidant activity would be associated with the presence in these plant species of phenolic acids and flavonoids. To our knowledge, there are no studies of the *in vitro* antioxidant activity of these species against hypochlorite. The latter, added to the common use of these plants in the folk medicine, led us to apply the PYR-based assay to study their hypochlorite removing capacity. Figure 7A shows the effect of the extract of *F. magellanica* on the consumption of PYR induced by hypochlorite. This extract protected PYR, as evidenced by the increase in fluorescence intensity measured in the plateau of the kinetic profiles (after 20 min incubation). In a similar way that experiments employing pure antioxidants, the protection of this plant extract did not change the initial consumption rate of PYR. This behavior, *i.e.*, effect on the fluorescence plateau without alter the initial rate of the reaction, was observed for all the samples studied. As is evidenced from data depicted in Figure 7B, increasing concentrations of extracts showed a linear relationship in agreement with Eqn 1. From these types of experiments were obtained PYR-HOCl values of the samples, which followed the order: *F. magellanica ≈ M. vulgare ≈ T. minuta > C. ambrosoides ≈ S. montana > T. praecox*.

**Figure 8:**
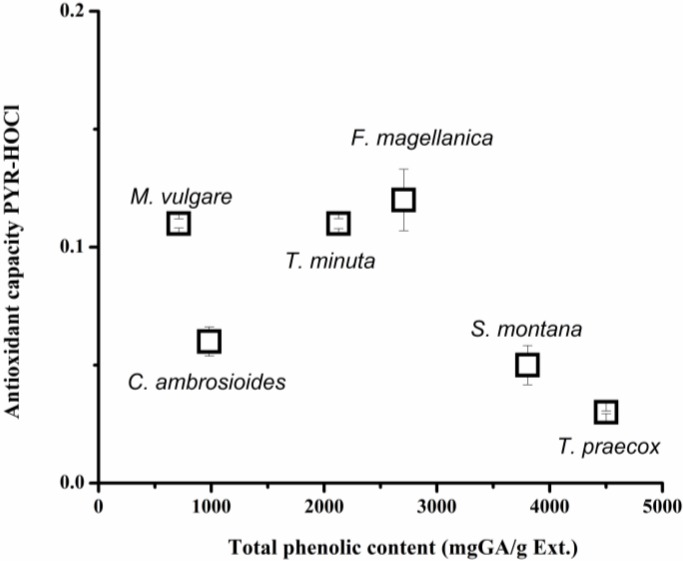
Dependence of the PYR-HOCl index with total phenol content of plant extracts.

The antioxidant capacity of plant extracts towards hypochlorite, estimated employing PYR as probe, show that the samples protected PYR to different degrees. This would imply that the samples contain different concentrations of antioxidants and/or that they contain compounds of different reactivity towards hypochlorite. To clarify this aspect we determined the total phenolic content of the samples by the Folin Ciocalteau assay. As presented in Figure 8, the PYR-HOCl values did not correlate with the total phenolic content of the samples. For example, the latter is evident if the results obtained employing *M. vulgare* and *T. praecox* extracts are analyzed. While the former showed, among all studied samples, one of the highest antioxidant activity, the latter extract presented the lowest PYR-HOCl value (Table 2). In contrast, the Folin Ciocalteau data showed that *T. praecox* extract had close to six times more phenols than *M. vulgare*. These results could be due to the fact that PYR-HOCl index is associated with the kinetic rate constant of the sample-hypochlorite reaction. Then, *M. vulgare* extract would contain antioxidants with higher reactivity towards hypochlorite than *T. praecox* extract.

**Table 2:** PYR-HOCl values (relative to gallic acid activity) of plant extracts. Data represent the mean of at least three independent experiments. Standard deviation (SD) represented less than 10% of the mean.

Plant Extracts	PYR-HOCl
*M. vulgare*	0.11
*T. minuta*	0.11
*C. ambrosioides*	0.06
*T. praecox*	0.03
*F. magellanica*	0.12
*S. montana*	0.05

## 3. Experimental

### 3.1. Chemicals

Trolox (6-hydroxy-2,5,8-tetramethylchroman-2-carboxylic acid), pyrogallol red (pyrogallol-sulfophtalein, PGR), pyranine (8-hydroxypyrene-1,3,6-trisulfonate trisodium salt, PYR), and all the antioxidants studied were purchased from Sigma-Aldrich (St. Louis, MO, USA). Hypochlorite and Folin-Ciocalteau reagent were supplied by Merck (Darmstadt, Germany). All compounds were employed as received.

### 3.2. Solutions

PGR and PYR stock solutions (1 mM) were prepared daily in Chelex-treated phosphate buffer 75 mM, pH 7.4. Hypochlorite stock solution (0.6 mM) was prepared daily by diluting commercial sodium hypochlorite with ultrapure water. This solution was kept in an ice bath and protected from light. Hypochlorite concentration was determined spectrophotometrically in 0.01 M NaOH using an extinction coefficient of 350 M^−1^cm^−1^at 292 nm [8]. Stock solutions of all antioxidants (10 mM) were prepared in ethanol. In all cases ethanol concentration did not excess 5% of the final working solution volume. Working solutions were prepared as follows: to a solution containing PGR (15 µM) or PYR (5 µM) an aliquot of hypochlorite (30 µL) was added to obtain a final hypochlorite concentration of 10 µM. Similar experiments were carried out in the presence of pure antioxidants (between 10 and 500 μM final concentrations) or dried extracts (between 0.1 and 40 μg/mL final concentrations). The consumption of PGR was evaluated from the progressive absorbance decrease measured at 540 nm in a thermostatized cuvette of a Hewlett Packard 8453 (Palo Alto, CA, USA) UV-visible spectrophotometer. The PYR consumption was assessed from the decrease in the sample fluorescence intensity (excitation: 460 nm; emission: 510 nm). Fluorescence measurements were carried out in a Perkin Elmer LS-55 spectrofluorimeter (Beaconsfield, UK).

### 3.3. Plant Material

Aerial parts of *Satureja montana* L; *Fuchsia magellanica* Lam.; *Thymus praecox* subsp. *arcticus* Jalas; *Marrubium vulgare* L.; *Tagetes minuta* L. and *Chenopodium ambrosioides* L. were grown, identified and collected in October 2010 in the Herbarium nursery, Peñalolén, Santiago, Chile. Plant material was dried at room temperature and powdered. Crude extracts were obtained by static maceration (2 × 24 h) of the dry material with methanol at room temperature. After extraction, the solvent was evaporated to dryness under reduced pressure.

### 3.4. Total Phenolics

Total phenol content in the extracts was determined according to the Folin–Ciocalteau colorimetric method [35,36]. Briefly, appropriate dilutions of the samples were incubated with 0.2 N Folin-Ciocalteau reagent (Merck, 2N, diluted ten-fold). After 5 min, sodium carbonate (75 g/L) was added. The mixtures were incubated for 60 min and the absorbance of the resulting blue color measured at 765 nm using an Agilent 8453 UV-visible spectrophotometer (Palo Alto, CA, USA). Quantification was done on the basis of a standard curve of gallic acid, and the results were expressed as milligrams of gallic acid equivalents per gram of dry extract.

### 3.5. Data Expression and Analysis

All data represent the mean values of at least three independent experiments, each conducted in triplicate. The standard deviations (SD) of such values are not included as these generally represent less than 10% of the means. The kinetic profiles and data treatment (linear regression analyses) were performed employing Origin 6.0 software. 

## 4. Conclusions

The results presented in this work show that PGR and PYR react efficiently with hypochlorite. Based on kinetic data and on the results from the protection afforded by pure antioxidants, we selected PYR as a better hypochlorite-oxidable probe than PGR. The use of PYR as probe allowed us to determine an antioxidant capacity index of plant extracts related to the reactivity of the samples towards hypochlorite. Plant extracts protected PYR giving a PYR-HOCl index that follows the order: *Fuchsia magellanica ≈ Marrubium vulgare ≈ Tagetes minuta > Chenopodium ambrosoides ≈ Satureja montana > Thymus praecox*.
